# SARS-CoV-2 infection in schools in a northern French city: a retrospective serological cohort study in an area of high transmission, France, January to April 2020

**DOI:** 10.2807/1560-7917.ES.2021.26.15.2001695

**Published:** 2021-04-15

**Authors:** Arnaud Fontanet, Laura Tondeur, Rebecca Grant, Sarah Temmam, Yoann Madec, Thomas Bigot, Ludivine Grzelak, Isabelle Cailleau, Camille Besombes, Marie-Noëlle Ungeheuer, Charlotte Renaudat, Blanca Liliana Perlaza, Laurence Arowas, Nathalie Jolly, Sandrine Fernandes Pellerin, Lucie Kuhmel, Isabelle Staropoli, Christèle Huon, Kuang-Yu Chen, Bernadette Crescenzo-Chaigne, Sandie Munier, Pierre Charneau, Caroline Demeret, Timothée Bruel, Marc Eloit, Olivier Schwartz, Bruno Hoen

**Affiliations:** 1 Emerging Diseases Epidemiology Unit, Institut Pasteur, Paris, France; 2 PACRI Unit, Conservatoire National des Arts et Métiers, Paris, France; 3 Pathogen Discovery Laboratory, Department of Virology, Institut Pasteur, Paris, France; 4 Bioinformatic and Biostatistic Hub – Computational Biology Department, Institut Pasteur, Paris, France; 5 Virus and Immunity Unit, Department of Virology, Institut Pasteur, Paris, France; 6 UMR 3569, Centre National de la Recherche Scientifique, Paris, France; 7 Université de Paris, Sorbonne Paris Cité, Paris, France; 8 Direction de la recherche médicale, Institut Pasteur, Paris, France; 9 ICAReB platform (Clinical Investigation & Access to Research Bioresources) of the Center for Translational Science, Institut Pasteur, Paris, France; 10 Center for Translational Sciences, Institut Pasteur, Paris, France; 11 Medical Center of the Institut Pasteur, Institut Pasteur, Paris, France; 12 Vaccine Research Institute, Creteil, France; 13 RNA Biology of Influenza Virus, Department of Virology, Institut Pasteur, Paris, France; 14 Molecular Genetics of RNA Viruses, Institut Pasteur, Paris, France; 15 Laboratoire Commun Pasteur/TheraVectys, Institut Pasteur, Paris, France; 16 Unité de Virologie Moléculaire et Vaccinologie, Institut Pasteur, Paris, France; 17 National Veterinary School of Alfort, Maisons-Alfort, France

**Keywords:** Coronavirus disease 2019 (COVID-19), SARS-CoV-2, Emerging infectious diseases, Epidemiology

## Abstract

**Background:**

Children’s role in SARS-CoV-2 epidemiology remains unclear. We investigated an initially unnoticed SARS-CoV-2 outbreak linked to schools in northern France, beginning as early as mid-January 2020.

**Aims:**

This retrospective observational study documents the extent of SARS-CoV-2 transmission, linked to an affected high school (n = 664 participants) and primary schools (n = 1,340 study participants), in the context of unsuspected SARS-CoV-2 circulation and limited control measures.

**Methods:**

Between 30 March and 30 April 2020, all school staff, as well as pupils and their parents and relatives were invited for SARS-CoV-2 antibody testing and to complete a questionnaire covering symptom history since 13 January 2020.

**Results:**

In the high school, infection attack rates were 38.1% (91/239), 43.4% (23/53), and 59.3% (16/27), in pupils, teachers, and non-teaching staff respectively vs 10.1% (23/228) and 12.0% (14/117) in the pupils’ parents and relatives (p < 0.001). Among the six primary schools, three children attending separate schools at the outbreak start, while symptomatic, might have introduced SARS-CoV-2 there, but symptomatic secondary cases related to them could not be definitely identified. In the primary schools overall, antibody prevalence in pupils sharing classes with symptomatic cases was higher than in pupils from other classes: 15/65 (23.1%) vs 30/445 (6.7%) (p < 0.001). Among 46 SARS-CoV-2 seropositive pupils < 12 years old, 20 were asymptomatic. Whether past HKU1 and OC43 seasonal coronavirus infection protected against SARS-CoV-2 infection in 6–11 year olds could not be inferred.

**Conclusions:**

Viral circulation can occur in high and primary schools so keeping them open requires consideration of appropriate control measures and enhanced surveillance.

## Introduction

As the coronavirus disease (COVID-19) pandemic continues to evolve, the extent of severe acute respiratory syndrome coronavirus 2 (SARS-CoV-2) infection in children has not been well documented and the role children may play in virus transmission remains unclear. During the first epidemic peak, many countries included school closures among the measures implemented to limit viral transmission, largely based on the evidence of the impact of school closures on influenza transmission [1]. As many schools have reopened or are now reopening, it is critical to evaluate the risk of viral circulation among pupils and staff in schools.

Initial epidemiological data from China indicated that children were significantly less affected by COVID-19 than adults, whether considering the total number of clinical cases, disease severity or fatal outcomes [2]. Similar findings have also been reported in other countries [3,4]. It is understood that children, when infected, present with mild and asymptomatic forms of the disease more frequently than adults, with severe and fatal outcomes remaining rare in children [5,6].

Younger children (≤ 10 years old) are generally believed to be less susceptible to SARS-CoV-2 infection than adults [7,8], and, in households, infections in such children usually originate from an older member [9]. Some studies have nevertheless documented similar secondary attack rates in families among children and adults [10]. In infected children, SARS-CoV-2 can be detected in the throat for 9–11 days after a positive PCR result [9] and for up to 1 month in faecal samples [11], with live virus culture from faecal samples rarely being successful [12]. Viral loads have been found to be similar between infected children and adults [13,14], suggesting that children could be as infectious as adults [15]. Nevertheless, because of the fewer and milder symptoms that children experience, transmission might be less efficient in this group.

At the time of school reopening at the beginning of the 2020/21 academic year in the northern hemisphere, the number of SARS-CoV-2 secondary transmissions in school settings documented in the scientific literature was limited. A meta-analysis of nationwide contact tracing data, including some in the school environment in Taiwan had found low secondary attack rates [16]. Very few or no secondary COVID-19 cases had been reported from investigations in Australia [17], France [18], Ireland [19], Singapore [20], the United Kingdom (UK) [21] and the United States (US) [22]. Exceptions, however, included important clusters in a high school in Israel after school reopening in May 2020 [23], and a large school community outbreak in Santiago, Chile in March 2020 [24].

The first three imported COVID-19 cases identified in France were reported on 24 January 2020 in travellers returning from Wuhan, China [25], but widespread autochthonous circulation of the virus was not reported until end of February 2020. On 24 February, a patient from the Hauts-de-France region, north of Paris, was admitted to hospital in Paris in a critical condition and was diagnosed with SARS-CoV-2 infection on 25 February 2020 (data not shown). The ensuing epidemiological investigation led to the identification of a cluster of COVID-19 that involved a high school in a small city (15,000 inhabitants), north of Paris (data not shown). Following this initial investigation, we conducted a retrospective closed cohort study to estimate the SARS-CoV-2 infection attack rate (IAR) in the high school and across primary schools in the same city using serological assays with high sensitivity and specificity for the detection of SARS-CoV-2 antibodies [26,27].

## Methods

After the confirmation of the case of COVID-19 from the Hauts-de-France region on 24 February 2020, an initial retrospective epidemiological investigation identified two teachers from the high school who had had symptoms consistent with COVID-19 on 2 February 2020. Since there was no known circulation of SARS-CoV-2 at that time in the region, no public health or social measures intended to limit the transmission of the virus had been implemented and no active SARS-CoV-2 testing was being conducted. A preliminary rapid investigation among adults and pupils who had respiratory symptoms and who were willing to be tested at the high school on 5–6 March 2020 revealed that 11/66 (16.7%) adults and 2/24 (8.3%) pupils had acute infection, as determined by a positive real-time reverse transcription (RT)-PCR test result.

### Study design

To further understand the extent of transmission in the high school, irrespective of symptoms, a retrospective closed cohort study was conducted between 30 March and 4 April 2020. All pupils – high schools in France usually provide education to children between 15 and 18 years-old – as well as teachers and non-teaching staff (administrative, cleaners, catering) from the high school were invited to participate in the investigation.

One month later (28–30 April 2020), to check if there might have been SARS-CoV-2 circulation in primary schools as well, a similar investigation was performed in all six primary schools – for children aged 6 to 11 years – of the same city. Again, all pupils, as well as teachers and non-teaching staff (administrative, cleaners, catering) from each of the six primary schools were invited to participate in the investigation. For each pupil, at least one parent was invited to participate in the study, as well as any of the other children over the age of 5 years of the household.

Following informed consent, all high school and primary school participants completed a questionnaire, which sought to obtain sociodemographic information, underlying medical conditions, history of symptoms since 13 January 2020, a date corresponding to approximately 2 weeks before the first clinical cases were reported in France [25], and history of COVID-19 diagnosis confirmed by RT-PCR, before the investigation. A 5 mL blood sample was taken from all participants, irrespective of whether they had reported fever or respiratory symptoms since 13 January 2020.

### Laboratory analyses

In the high school study, all serum samples were tested for antibody responses to SARS-CoV-2 using several assays developed by Institut Pasteur: an ELISA N assay, detecting antibodies binding to the nucleocapsid (N) protein; a S-Flow assay, which is a flow-cytometry based assay detecting anti-spike (S) IgG; and a luciferase immunoprecipitation system (LIPS) assay, which is an immunoprecipitation-based assay detecting anti-N, anti-S1 and anti-S2 IgG. Cut-offs were chosen so that the specificity, based on the evaluation performed on sera from 240 pre-epidemic blood donors, would be higher than 99% for each of the tests [26]. In the high school study, participants were considered seropositive for SARS-CoV-2 antibodies if any of the serological assay tests were positive. In a further study, the S-Flow assay was shown to have a sensitivity of 99.4% to detect mild forms of COVID-19, which had been RT-PCR-confirmed [27]. As such, for the primary schools’ study, only the S-Flow was used for first line serological testing. All sera for the S-Flow assay were tested at a 1:300 dilution.

Samples were also tested for neutralisation activity using a viral pseudotype-based assay. Briefly, single cycle lentiviral pseudotypes coated with the S protein and encoding for a luciferase reporter gene were pre-incubated with the serum to be tested at a dilution of 1:100 for the high school sera, and 1:40 for the primary school sera, and added to 293T-angiotensin-converting enzyme 2 (ACE2) target cells [28]. The luciferase signal was measured after 48 hours. The percentage of neutralisation activity was calculated by comparing the signal obtained with each serum to the signal generated by control negative sera.

In addition, in a subgroup of samples (see below), the LIPS assay was used to assess antibody responses to the full S ectodomain in a pre-fusion conformation of the SARS-CoV-2, the two seasonal human beta-coronaviruses (HKU1, OC43) and one seasonal alpha-coronavirus (229E). Technical details and sensitivity and specificity information of the assay are available elsewhere [28].

### Case definitions

Any participant with a positive serology at the time of blood sampling was considered as being SARS-CoV-2 seropositive. Seropositive individuals were categorised as symptomatic cases if any symptoms were reported by the participant since 13 January 2020, or, alternatively, as asymptomatic. As the clinical presentation of COVID-19 was not well characterised at the time the study was conducted, there was no restriction on symptoms. Symptoms were considered only if they occurred at least 7 days before the date of blood sample collection to allow time for seroconversion [29]. Symptoms were further categorised as major (fever, dry cough, dyspnoea, anosmia and ageusia) or minor (sore throat, rhinitis, myalgia, diarrhoea, headache, asthenia).

### Statistical analyses

The IAR was defined as the proportion of all participants with SARS-CoV-2 antibodies detected, which was used as a proxy for prior SARS-CoV-2 infection. Binomial exact confidence intervals (CI) were calculated for proportions. IAR was compared according to age, sex, occupation, comorbid conditions and recent symptoms using a chi-squared test. Positive predictive values were calculated for symptoms potentially associated with the detection of SARS-CoV-2 antibodies. Antibody levels against seasonal human coronaviruses (HCoVs) were compared using a Wilcoxon matched pairs signed rank sum test in a subgroup derived from the study population of children with neutralising antibodies (n = 49) matched for age and sex with children without neutralising antibodies (n = 98). All statistical analyses were performed using Stata 15.0 (StataCorp, College Station, Texas, US).

### Ethical statement

This study was registered with ClinicalTrials.gov (NCT04325646) and received ethical approval by the Comité de Protection des Personnes Ile de France III. Informed consent was obtained from all participants, and parents provided informed consent for any children under the age of 18 years.

## Results

### Evidence of SARS-CoV-2 circulation in the schools

From 30 March to 4 April 2020, 878 of 1,262 high school pupils, teachers, and non-teaching staff were invited by email to participate in the investigation (email addresses were not available for 384). Of these, 323 (36.8%) responded and participated in the study: 243 pupils, 53 teachers and 27 non-teaching staff. In addition, 348 parents of the 243 pupils and their relatives living in the same household joined the study (see Figure S1). Blood samples could not be obtained from seven, making a total high school study population of 664 study participants. Pupils (n = 239) and their parents (n = 228) constituted the majority of the study population (36.0% and 34.3%, respectively) (see Table S1).

**Figure 1:**
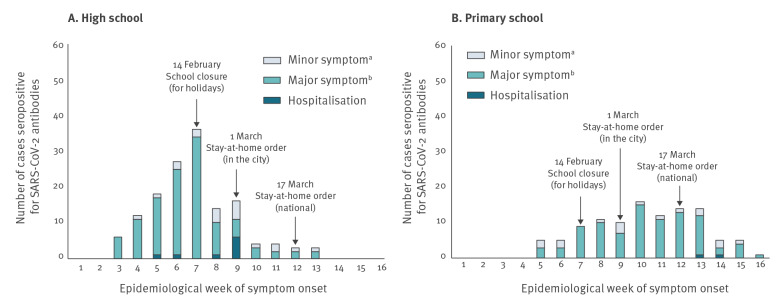
Timeline of symptom onset among (A) 143 symptomatic cases who were seropositive for SARS-CoV-2 antibodies in a high school and (B) 107 symptomatic cases who were seropositive for SARS-CoV-2 antibodies in primary schools, investigation in a city in northern France, 13 January–19 April 2020 (n = 250)

The overall IAR among those linked to the high school was 25.1% (167/664). Table 1 shows the proportion of those linked to the high school with SARS-CoV-2 antibodies (see Table S2 for the test-specific serological results). The IAR was higher (38.1%, 43.4%, and 59.3% in pupils, teachers, and non-teaching staff, respectively) than in parents and relatives (10.1% and 12.0%, respectively) (p < 0.001). More specifically, the IAR was 5.3% among parents of a non-infected pupil and 18.3% among those of an infected pupil (p = 0.002). The IAR was 3.1% among relatives of a non-infected pupil and 20.0% among relatives of an infected pupil (p = 0.005). Figure 1 displays, for both types of schools, the distributions by week of symptom onset of seropositive cases. In the high school (Figure 1A), a total of 143 such cases were observed. From 13 January 2020, the weekly occurrences of symptomatic cases increased rapidly until school closure for the holidays on 14 February (end of week 7). Subsequently, the number of new cases dropped, with a more pronounced decrease after the introduction of local movement restriction measures, including stay-at-home orders, in the Hauts-de-France region on 1 March.

**Table 1:** Infection attack rates among participants of investigations documenting the extent of SARS-CoV-2 transmission in a high school and primary schools, northern France, 30 March–30 April 2020 (n = 2,004 participants)

Characteristic		High school investigation (n = 664)				Primary school investigation (n = 1,340)			
		Total number	Number of seropositive	Per cent seropositive	p	Total number	Number of seropositive	Per cent seropositive	p
**Sex**									
Male		253	54	21.3	0.08	571	54	9.5	0.34
Female		411	113	27.5		769	85	11.1	
**Age group in years**									
< 12		8	0	0.0	< 0.001	538	46	8.6	0.10
12–17		235	82	34.9		78	12	15.4	
≥ 18		421	85	20.2		724	81	11.2	
**Type of participant**									
Pupil		239	91	38.1	< 0.001	510	45	8.8	0.36
Teacher		53	23	43.4		41	3	7.3	
Non-teaching staff		27	16	59.3		28	1	3.6	
Parents	All	228	23	10.1		642	76	11.8	
	Of an infected pupil	82	15	18.3		59	36	61.0	
	Of a non-infected pupil	132	7	5.3		569	39	6.9	
	Other	14	1	7.1		14	1	7.1	
Relatives	All	117	14	12.0		119	14	11.8	
	Of an infected pupil	50	10	20.0		9	4	44.4	
	Of a non-infected pupil	65	2	3.1		107	10	9.3	
	Other	2	2	100.0		3	0	0.0	

SARS-CoV-2: severe acute respiratory syndrome coronavirus 2.

SARS-CoV-2: severe acute respiratory syndrome coronavirus 2.

^a^ Major symptoms included ageusia and anosmia, fever, dry cough and dyspnoea.

^b^ Minor symptoms included asthenia, diarrhoea, headache, myalgia, rhinitis, sore throat.

For the primary schools, 1,047 pupils and 51 teachers from six primary schools were invited by email to participate in the investigation between 28 and 30 April 2020. Of these, 541 (51.7%) pupils and 46 (90.2%) teachers accepted to participate in the study. Thirty-one pupils were excluded as they refused phlebotomy, as were four teachers not directly affiliated with any of the six schools. This resulted in 510 pupils and 42 teachers with a blood sample to be analysed. In addition, 641 parents of pupils, 119 relatives of pupils sharing the same household, and 28 non-teaching staff completed the study population (Figure S2). Table S3 indicates the characteristics of the 1,340 participants.

The overall IAR across study participants linked to primary schools was 139/1,340 (10.4%). It did not differ by sex, age categories, or type of participants (Table 1). Parents of infected pupils had higher IAR compared with parents of non-infected pupils (61.0% vs 6.9%; p < 0.0001), and relatives of infected pupils had higher IAR compared with relatives of non-infected pupils (44.4% vs 9.1%; p = 0.002) (Table 1). The epidemic curve, based on week of symptom onset experienced by the 107 SARS-CoV-2 symptomatic cases, increased up to the week ending 8 March (week 10), followed by a sustained decline (Figure 1B).

Based on the presence and timing of symptoms among seropositive children, we could identify three instances of introduction of infected symptomatic children in three separate schools before the closure of the schools for holidays on 14 February (end of week 7) (see Supplementary material text and Figure S3). We were only able to identify one symptomatic secondary case among pupils, teachers and non-teaching staff epidemiologically linked to one of the three infected symptomatic children – a teacher (school C) who had onset of symptoms on 12 February, but who also had a close contact (meal at a restaurant) with a RT-PCR confirmed COVID-19 case outside of the school 5 days before becoming unwell. However, antibody prevalence across the primary school pupils was higher among those attending the classes with symptomatic seropositive cases compared with those in other classes: 15/65 (23.1%) vs 30/445 (6.7%) (p < 0.001). Table 2 shows the proportion of children with SARS-CoV-2 antibodies by school and by class.

**Table 2:** Proportion of pupils with SARS-CoV-2 antibodies by class and by primary school in a city in northern France, 13 January–19 April 2020 (n = 510)

School	Class and proportion of pupils with SARS-CoV-2 antibodies									All classes	
	CP	CP/CE1^a^	CE1	CE1/CE2^a^	CE2	CE2/CM1^a^	CM1	CM1/CM2^a^	CM2		
	Proportion	Proportion	Proportion	Proportion	Proportion	Proportion	Proportion	Proportion	Proportion	Proportion	%
A	1/7	0/8	0/7	NA	0/9	NA	0/10	1/12	0/8	2/61	3.3
B	2/17	0/1	NA	1/32	NA	3/18	NA	5/22	NA	11/90	12.2
C	0/12	NA	3/10	NA	1/14	NA	NA	10/32	NA	14/68	20.6
D	0/8	0/8	1/13	0/9	1/11	NA	1/24	0/1	1/12	4/87^b^	4.6
E	0/12	2/28	NA	0/9	2/15	NA	2/15	0/25	0/13	6/117	5.1
F	0/16	NA	1/11	NA	2/18	NA	4/22	NA	1/20	8/87	9.2

CE1: cours élémentaire première année; CE2: cours élémentaire deuxième année; CM1: cours moyen première année; CM2: cours moyen deuxième année; CP: cours préparatoire; NA: not applicable (the school in question had few pupils so there were no children populating the class in question); SARS-CoV-2: severe acute respiratory syndrome coronavirus 2.

The classes CP, CE1, CE2, CM1, CM2 respectively correspond to the first to fifth years (levels) of primary school. Shaded areas: classes with one documented symptomatic introduction.

^a^ These are classes combining two levels.

^b^ Class missing for one child.

### Symptoms of SARS-CoV-2-infected children, teenagers and adults

Symptoms associated with SARS-CoV-2 infection differed according to age groups (Table 3). Some associations had only borderline statistical significance and should be considered cautiously, taking into account that no correction was performed for multiple testing. Among adults, fever, cough, dyspnoea, ageusia, anosmia, myalgia, sore throat, rhinorrhoea, headache, asthenia, nausea, and diarrhoea, were all positively associated with SARS-CoV-2 infection, with high positive predictive values for ageusia (80.8%) and anosmia (89.4%). Among teenagers (12–17 years), fever, ageusia, anosmia, and diarrhoea were positively associated with SARS-CoV-2 infection, with high positive predictive values for ageusia (89.5%) and anosmia (80.0%). Among children less than 12 years of age, only asthenia (marginally, p = 0.06), and diarrhoea were positively associated with SARS-CoV-2 infection, and no symptom had any relevant positive predictive value. The rate of hospitalisation was 0% (0/46;  one-sided 97.5% CI: 0–7.7%) among the less than 12 years; 2.1% (2/94; 95% CI: 0.3–7.5%) among the 12–17 years; and 5.4% (9/166; 95% CI: 2.5–10.0%) among adults. There were no deaths. Across the study period, among those who were seropositive, 20/46 (43.5%) children aged less than 12 years, 22/94 (23.4%) 12–17 years, and 14/166 (8.4%) adults reported no symptoms (p < 0.001). Symptoms of respiratory infections – fever, cough, rhinitis – were common among the participants without SARS-CoV-2 antibodies during the study period.

**Table 3:** SARS-CoV-2 infection attack rates (%) by symptoms and age category in schools in a city in northern France, 13 January–19 April 2020 (n = 2,004 participants)

Symptoms	Children < 12 years (n = 546)					Children 12–17 years (n = 313)					Adults (n = 1,145)				
	Number of individuals	Proportion with the symptom (%)	Number infected	Proportion with the symptom infected (%)	p value	Number of individuals	Proportion with the symptom (%)	Number infected	Proportion with the symptom infected (%)	p value	Number of individuals	Proportion with the symptom (%)	Number infected	Proportion with the symptom infected (%)	p value
**Fever**															
Yes	121	22.2	13	10.7	0.30	84	26.8	33	39.3	0.03	254	22.2	81	31.9	< 0.001
No	425	77.8	33	7.8		229	73.2	61	26.6		891	77.8	85	9.5	
**Cough**															
Yes	124	22.7	8	6.5	0.37	105	32.9	32	30.5	0.90	349	30.5	87	24.9	< 0.001
No	422	77.3	38	9.0		208	66.5	62	29.8		796	69.5	79	9.9	
**Dyspnoea**															
Yes	20	3.7	3	15.0	0.28	33	10.5	5	15.1	0.05	157	13.7	49	31.2	< 0.001
No	526	96.3	43	8.2		280	89.5	89	31.8		988	86.3	117	11.8	
**Ageusia**															
Yes	4	0.7	0	0	0.99	19	6.1	17	89.5	< 0.001	94	8.2	76	80.9	< 0.001
No	542	99.3	46	8.5		294	93.9	77	26.2		1,051	91.7	90	8.6	
**Anosmia**															
Yes	2	0.4	0	0	0.99	20	6.4	16	80.0	< 0.001	85	7.4	76	89.4	< 0.001
No	544	99.6	46	8.5		293	93.6	78	26.6		1,060	92.6	90	8.5	
**Myalgia**															
Yes	40	7.3	4	10.0	0.71	55	17.6	19	34.5	0.42	268	23.4	83	31.0	< 0.001
No	506	92.7	42	8.3		258	82.4	75	29.1		877	76.6	83	9.5	
**Sore throat**															
Yes	90	16.5	8	8.9	0.86	87	27.8	25	28.7	0.76	261	22.8	53	20.3	0.002
No	456	83.5	38	8.3		226	72.2	69	30.5		884	77.2	113	12.8	
**Rhinorrhoea**															
Yes	97	17.8	10	10.3	0.46	114	36.4	41	36.0	0.08	283	24.7	62	21.9	< 0.001
No	449	82.2	36	8.0		199	63.5	53	26.6		862	75.3	104	12.1	
**Headache**															
Yes	101	18.5	8	7.9	0.84	91	29.1	31	34.1	0.32	332	29.0	84	25.3	< 0.001
No	445	81.5	38	8.5		222	70.9	63	28.4		813	71.0	82	10.1	
**Asthenia**															
Yes	79	14.4	11	13.9	0.06	77	24.6	26	33.8	0.41	350	30.6	95	27.1	< 0.001
No	467	85.5	35	7.5		236	75.4	68	28.8		795	69.4	71	8.9	
**Chest pain**															
Yes	0	0	0	0	NA	0	0	0	0	-	7	0.6	0	0	0.61
No	546	100	46	8.4		313	100	94	30.0		1,138	99.4	166	14.6	
**Nausea**															
Yes	3	0.5	1	33.3	0.23	3	1.0	2	66.7	0.22	10	0.9	4	40.0	0.04
No	543	99.5	45	8.3		310	99.0	92	29.7		1,135	99.1	162	14.3	
**Vomiting**															
Yes	23	4.2	1	4.3	0.71	13	4.2	5	38.5	0.54	18	1.6	2	11.1	0.99
No	523	95.8	45	8.6		300	95.8	89	29.7		1,127	98.4	164	14.6	
**Abdominal pain**															
Yes	6	1.1	0	0	0.99	2	0.6	0	0	0.99	12	1.0	4	33.3	0.08
No	540	98.9	46	8.5		311	99.4	94	30.2		1,133	99.0	162	14.3	
**Diarrhoea**															
Yes	50	9.2	9	18.0	0.01	37	11.8	17	45.9	0.03	151	13.2	38	25.2	< 0.001
No	496	90.8	37	7.5		276	88.2	77	27.9		994	86.8	128	12.9	
**Symptom severity**															
None	274	50.2	20	7.3	0.62	110	35.1	22	20.0	0.01	429	37.5	14	3.3	< 0.001
Minor^a^ only	89	16.3	8	9.0		54	17.3	15	27.8		214	18.7	14	6.5	
Major^b^	183	33.5	18	9.8		149	47.6	57	38.3		502	43.8	138	27.5	
**Medical consultation^c^**															
Yes	99	36.4	8	8.1	0.53	44	21.7	17	38.6	0.62	255	35.6	73	28.6	< 0.001
No	173	63.6	18	10.4		159	78.3	55	34.6		461	64.4	79	17.1	
**Hospitalisation**															
Yes	1	0.2	0	0	0.99	4	1.3	2	50.0	0.59	14	1.2	9	64.3	< 0.001
No	545	99.8	46	8.5		309	98.7	92	29.8		1,131	98.8	157	13.9	

NA: not applicable.

^a^ Minor symptoms included asthenia, diarrhoea, headache, myalgia, rhinitis, and sore throat.

^b^ Major symptoms included ageusia and anosmia, fever, dry cough and dyspnoea.

**^c^** Only among participants who declared symptoms, including children < 12 years (n = 272); children 12–17 years (n = 203); adults (n = 716).

### Proportion of cases with neutralising antibodies

Results concerning antibodies with neutralising activity > 50% were available for 303 of the 306 seropositive study participants. Among these 303, neutralising antibodies were detected in 218 (72.0%) and were as common among children (105/148, 71.0%) as adults (113/155, 72.9%) (p > 0.05). Neutralising antibodies were higher in those who were symptomatic (184/246, 74.8%) compared to those who reported no symptoms (34/57, 59.6%; p=0.02). Neutralising antibodies were more common in infected participants with certain symptoms compared to those without the respective symptom, including ageusia (74/92, 80.4% vs 144/211, 68.3%; p = 0.03), anosmia (73/91, 80.2% vs 145/212, 68.4%; p=0.04), asthenia (108/132, 81.8%% vs 110/171, 64.3%; p =0.001), headache (97/121, 80.2% vs 121/182, 66.5%, p=0.009), diarrhoea (54/64, 84.4% vs 164/239, 68.6%; p =0.01), and myalgia (88/106, 83.0% vs 130/197, 66.0%; p = 0.002).

### Antibodies to seasonal human coronaviruses and SARS-CoV infection in children

**Figure 2:**
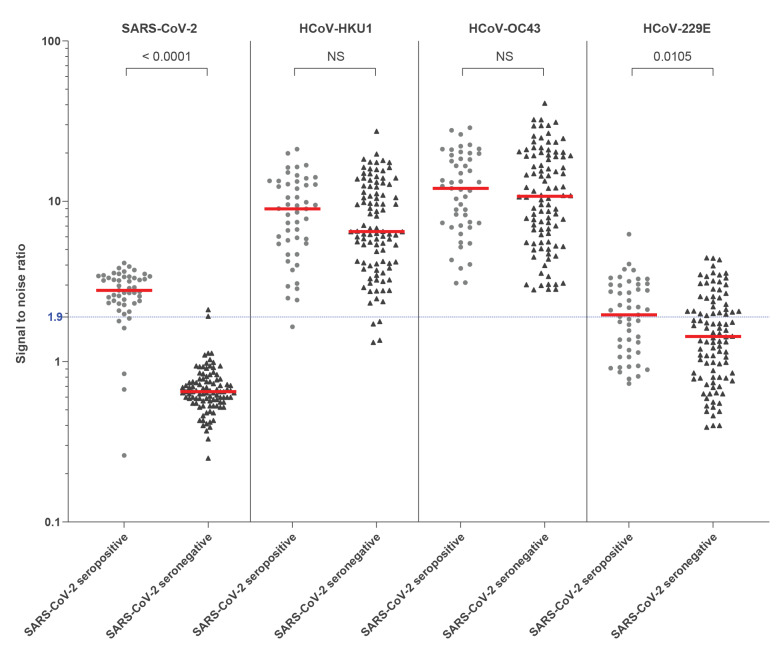
SARS-CoV-2 and seasonal HCoV antibody responses by LIPS in children with (n = 49) and without (n = 98) SARS-CoV-2 neutralising antibodies

HCoV: human coronaviruses; LIPS: luciferase immunoprecipitation system; NS: non significant; SARS-CoV-2: severe acute respiratory syndrome coronavirus 2.

We compared antibody levels against seasonal human coronaviruses (HCoVs) in a subpopulation of children aged 6–11 years with (n = 49) and without (n = 98) antibodies for SARS-CoV-2, matched for age and sex. Antibodies against seasonal betacoronaviruses HKU1 and OC43 were found at levels associated with past infection in 142/147 (96.6%) and 147/147 (100%) of children, respectively. Antibody levels to betacoronaviruses (HKU1 and OC43) were similar between SARS-CoV-2 seropositive and seronegative children, whereas antibody levels against alphacoronavirus (229E) were higher among SARS-CoV-2 seropositive compared with seronegative children (p = 0.01) (Figure 2).

The LIPS assay was used to assess antibody responses to the full spike ectodomain in a pre-fusion conformation of the SARS-CoV-2, the two seasonal human beta-coronaviruses (HKU1, OC43) and one seasonal alpha-coronavirus (229E). Technical details and sensitivity and specificity information of the assay are available elsewhere [28]. The blue horizontal line across the Figure at 1.9 represents the positivity threshold for antibody response. Medians are shown in red. The statistical significance of the difference in antibody response between SARS-CoV-2 seronegative and seropositive groups is indicated above each panel.

## Discussion

This comparative study in a region, which turned out to have high SARS-CoV-2 community transmission early in the COVID-19 pandemic in France [30], provides important information regarding the circulation of SARS-CoV-2 in the school environment and into the household setting. Viral circulation in schools and families of pupils in February 2020 took place at a time when no one was aware of the presence of the SARS-CoV-2 virus in the community, since it was only on 25 February that the first COVID-19 diagnosis was made in a patient from the region who was hospitalised in Paris. As a result, this study allowed us to document the circulation of the virus in the absence of control measures, at least until 25 February. First, while the high school experienced a noticeable outbreak of symptomatic infections, the circulation of the virus in the primary schools was partly silent, with a large proportion (43.5%) of asymptomatic infections among children under 12 years old. Second, parents and relatives of infected pupils were more likely to be infected compared with those of non-infected pupils, particularly for families of primary school pupils. Third, infected children less than 12 years of age experienced mild forms of disease, with no specific symptoms, or were asymptomatic, while teenagers and adults experienced similar forms of disease. Adults and children developed neutralising antibodies at a similar rate, with a higher proportion of those with neutralising antibodies among symptomatic compared with non-symptomatic participants. Finally, past infection with seasonal coronavirus HKU1 and OC43 was very common (>95% of primary school-aged children), precluding the possibility of studying any effect of past infection with these two coronaviruses on the risk of acquiring SARS-CoV-2 infection.

In the high school, where the virus was introduced in early February, an important outbreak took place, with 41% (130/319) of infected pupils and staff at the time of our investigation. School closure for the holidays had a clear impact on viral circulation. In primary schools, among symptomatic cases during the 3 weeks preceding the school closure for holidays (14 February) and then the stay at home order in the city (1 March), we could identify three SARS-CoV-2 infected pupils in three separate schools. We were only able to identify one symptomatic secondary case linked to one of the three infected symptomatic children – a teacher, but this case could have equally been infected by another SARS-CoV-2 infected person outside of the school. However, the prevalence of antibodies was higher among pupils attending the classes where cases were identified, suggesting silent circulation of the virus in these classes. These findings differ from results of previous studies [16-22], which all show limited secondary transmission in school settings or school aged children. Only studies in Israel [23] and Chile [24] have documented substantial outbreaks linked to school settings. The study in Chile used serologic testing like our study, but found lower antibody prevalence among high school pupils compared with younger pupils [24]. One likely explanation for the discordant findings of our study with respect to other published studies is the absence of infection prevention and control measures in classes at a time no one knew the virus was circulating, and the presence of children with symptoms in the classes. Together, these findings suggest that high school aged children have similar susceptibility to SARS-CoV-2 infection as adults in the school setting, and can transmit SARS-CoV-2 efficiently. For primary school aged children, the presence of symptoms in the children in each of the schools may have facilitated transmission to others, although the overall IAR in primary schools was low.

Since the two teachers from the high school diagnosed on 2 February seem to be among the first cases identified in the city, it is likely that the high school outbreak may have contributed to the early dissemination of the virus into the pupils’ homes. As a result, the increase in SARS-CoV-2 antibody prevalence among parents (from 5.3% to 18.3%) and relatives (from 3.1% to 20.0%) of non-infected compared with infected pupils may reflect the secondary intra-household attack rate after introduction of the virus into the homes. Interestingly, in the absence of knowledge of virus circulation and of control measures at that time, a relatively limited proportion of household members became infected. Further, these figures align well with the 15% secondary attack rate observed in a study of cases and close contacts in Shenzhen, China [10]. In contrast, in the primary schools, the high prevalence of antibodies among parents (61.0%) and relatives (44.4%) of infected pupils suggest that contacts between primary school aged children with parents and relatives may be closer compared with adolescents and parents, or that parents and relatives may have been the source of infection for the primary school pupils.

In adults, symptoms associated with SARS-CoV-2 infection were fever, cough, shortness of breath, ageusia, anosmia, headache, asthenia, myalgia, sore throat, and diarrhoea, all since known to be associated with COVID-19. Symptoms with the highest predictive values for SARS-CoV-2 infection were anosmia and ageusia, as previously reported [31,32]. Symptoms were less specific in children, with only fatigue and diarrhoea being associated with SARS-CoV-2 infection for primary school children. Altogether, in this community-based study, the rate of hospitalisation remained low, ranging from 0% in primary school children to 5.4% in adults. This study also gave an opportunity to estimate the proportion of asymptomatic infections, which increased from 8.4% in adults to 23.4% in high school children and 43.5% in primary school children. These figures are likely underestimated, since symptoms related to other respiratory infections may have been attributed to SARS-CoV-2 in seropositive individuals who may have been otherwise asymptomatic. Indeed, the current estimate of the proportion of individuals who are infected with SARS-CoV-2 and remain asymptomatic is ca 20%, with a suggestion that children have a higher proportion of asymptomatic infections [33].

Most (218/303, 72.0%) seropositive individuals had neutralising antibodies up to 3 months after the presumed start of the outbreak, with no difference between children and adults. Individuals with symptoms were more likely to have neutralising antibodies compared with individuals without, as described elsewhere [34]. T-cell reactivity against SARS-CoV-2 proteins in unexposed individuals has been identified as being attributable to cross-reactivity with past seasonal coronaviruses infection [35], and we investigated whether past seasonal coronaviruses infections may protect against SARS-CoV-2 infection. In this study, almost all children aged 6–11 years tested had previous immunity against human coronaviruses (HCoV) HKU1 and OC43, precluding the possibility of studying any effect of past infection with these two coronaviruses on the risk of acquiring SARS-CoV-2 infection. Interestingly, antibody levels against HCoV 229E were higher among SARS-CoV-2 infected compared with non-infected, raising the question of whether recent infection with SARS-CoV-2 may have had a boosting effect on past HCoV 229E antibodies. One previous study found no evidence of cross-protective immunity linked to previous infection by seasonal HCoVs against SARS-CoV-2 infection [28], while a different study suggests that SARS-CoV-2 antibodies reactive to the S protein of OC43 are boosted following SARS-CoV-2 infection [36].

The current study has several important limitations among which the particularly low rate of high school participants (only 37% of the population invited for the study came for the high school study). Since access to COVID-19 diagnosis was very limited until our investigations, it is unlikely that knowledge of SARS-CoV-2 infection status influenced decisions to join the study, and therefore the IAR estimates of our study. Although all pupils, staff and families of pupils were invited to participate in the study, we cannot exclude that people who were symptomatic during the study period may have been more likely to participate in the study than those who were not symptomatic. The overall IAR of 10.4% among participants other than those linked to the high school aligns well with the overall figure for high transmission areas of France after the first epidemic wave [37]. Further, the inferences as to where transmission may have occurred – in the school or in the home – are made more difficult among the primary school aged children through the use of serology for retrospective diagnosis and a large proportion of mild or asymptomatic infection. Nonetheless, the higher proportion of infected pupils in classes with symptomatic and infected children allows us to speculate that transmission likely occurred in the school setting.

### Conclusion

Our investigations identified SARS-CoV-2 circulation in both a high school and primary schools at the very early onset of the pandemic, in a context of unsuspected circulation in the community and absence of control measures. Decisions to reopen or close schools should be considered carefully in the context of the extent of transmission in the wider community. Ongoing monitoring for possible resurgence in infections would be needed, as well as strategies to limit transmission in the school setting, including testing of all those with symptoms, rapid isolation of cases and quarantine and testing of contacts, hand hygiene, physical distancing, respiratory etiquette, cohorting of classes, and the wearing of masks for older pupils. 

## Supplementary Data

598000 Supplementary Material

## References

[1] Cauchemez S Valleron AJ Boëlle PY Flahault A Ferguson NM. Estimating the impact of school closure on influenza transmission from Sentinel data. Nature. 2008;452(7188):750-4. 10.1038/nature06732 18401408

[2] Epidemiology Working Group for NCIP Epidemic Response, Chinese Center for Disease Control and Prevention. [The epidemiological characteristics of an outbreak of 2019 novel coronavirus diseases (COVID-19) in China]. Zhonghua Liu Xing Bing Xue Za Zhi. 2020;41(2):145-51. 32064853 10.3760/cma.j.issn.0254-6450.2020.02.003

[3] Bialek S Gierke R Hughes M McNamara LA Pilishvili T Skoff T CDC COVID-19 Response Team. Coronavirus Disease 2019 in Children - United States, February 12-April 2, 2020. MMWR Morb Mortal Wkly Rep. 2020;69(14):422-6. 10.15585/mmwr.mm6914e4 32271728 PMC7147903

[4] Riccardo F, Ajelli M, Andrianou X, et al. Epidemiological characteristics of COVID-19 cases in Italy and estimates of the reproductive numbers one month into the epidemic. Medrxiv 2020; doi: 10.1101/2020.04.08.20056861. PMC7730489 33303064

[5] Dong Y Mo X Hu Y Qi X Jiang F Jiang Z Epidemiology of Covid-19 among children in China. Pediatrics. 2020;145(6):e20200702. 10.1542/peds.2020-0702 32179660

[6] Shekerdemian LS Mahmood NR Wolfe KK Riggs BJ Ross CE McKiernan CA International COVID-19 PICU Collaborative. Characteristics and Outcomes of Children With Coronavirus Disease 2019 (COVID-19) Infection Admitted to US and Canadian Pediatric Intensive Care Units. JAMA Pediatr. 2020;174(9):868-73. 10.1001/jamapediatrics.2020.1948 32392288 PMC7489842

[7] Viner RM Mytton OT Bonell C Melendez-Torres GJ Ward J Hudson L Susceptibility to SARS-CoV-2 Infection Among Children and Adolescents Compared With Adults: A Systematic Review and Meta-analysis. JAMA Pediatr. 2021;175(2):143-56. 10.1001/jamapediatrics.2020.4573 32975552 PMC7519436

[8] Stringhini S Wisniak A Piumatti G Azman AS Lauer SA Baysson H Seroprevalence of anti-SARS-CoV-2 IgG antibodies in Geneva, Switzerland (SEROCoV-POP): a population-based study. Lancet. 2020;396(10247):313-9. 10.1016/S0140-6736(20)31304-0 32534626 PMC7289564

[9] Qiu H Wu J Hong L Luo Y Song Q Chen D. Clinical and epidemiological features of 36 children with coronavirus disease 2019 (COVID-19) in Zhejiang, China: an observational cohort study. Lancet Infect Dis. 2020;20(6):689-96. 10.1016/S1473-3099(20)30198-5 32220650 PMC7158906

[10] Bi Q Wu Y Mei S Ye C Zou X Zhang Z Epidemiology and transmission of COVID-19 in 391 cases and 1286 of their close contacts in Shenzhen, China: a retrospective cohort study. Lancet Infect Dis. 2020;20(8):911-9. 10.1016/S1473-3099(20)30287-5 32353347 PMC7185944

[11] Jiehao C Jin X Daojiong L Zhi Y Lei X Zhenghai Q A Case series of children with 2019 novel coronavirus infection: clinical and epidemiological features. Clin Infect Dis. 2020;71(6):1547-51. 10.1093/cid/ciaa198 32112072 PMC7108143

[12] Cevik M Tate M Lloyd O Maraolo AE Schafers J Ho A. SARS-CoV-2, SARS-CoV, and MERS-CoV viral load dynamics, duration of viral shedding, and infectiousness: a systematic review and meta-analysis. Lancet Microbe. 2021;2(1):e13-22. 10.1016/S2666-5247(20)30172-5 33521734 PMC7837230

[13] Jones TC, Mühlemann B, Veith T, et al. An analysis of SARS-CoV-2 viral load by patient age. https://zoonosen.charite.de/fileadmin/user_upload/microsites/m_cc05/virologie-ccm/dateien_upload/Weitere_Dateien/analysis-of-SARS-CoV-2-viral-load-by-patient-age.pdf

[14] L’Huillier AG Torriani G Pigny F Kaiser L Eckerle I. Culture-Competent SARS-CoV-2 in Nasopharynx of Symptomatic Neonates, Children, and Adolescents. Emerg Infect Dis. 2020;26(10):2494-7. 10.3201/eid2610.202403 32603290 PMC7510703

[15] Patel AB Verma A. Nasal ACE2 Levels and COVID-19 in Children. JAMA. 2020;323(23):2386-7. 10.1001/jama.2020.8946 32432681

[16] Huang YT Tu YK Lai PC. Estimation of the secondary attack rate of COVID-19 using proportional meta-analysis of nationwide contact tracing data in Taiwan. J Microbiol Immunol Infect. 2021;54(1):89-92. 10.1016/j.jmii.2020.06.003 32553448 PMC7289119

[17] Macartney K Quinn HE Pillsbury AJ Koirala A Deng L Winkler N NSW COVID-19 Schools Study Team. Transmission of SARS-CoV-2 in Australian educational settings: a prospective cohort study. Lancet Child Adolesc Health. 2020;4(11):807-16. 10.1016/S2352-4642(20)30251-0 32758454 PMC7398658

[18] Danis K Epaulard O Bénet T Gaymard A Campoy S Botelho-Nevers E Investigation Team. Cluster of coronavirus disease 2019 (COVID-19) in the French Alps, February 2020. Clin Infect Dis. 2020;71(15):825-32. 10.1093/cid/ciaa424 32277759 PMC7184384

[19] Heavey L Casey G Kelly C Kelly D McDarby G. No evidence of secondary transmission of COVID-19 from children attending school in Ireland, 2020. Euro Surveill. 2020;25(21):2000903. 10.2807/1560-7917.ES.2020.25.21.2000903 32489179 PMC7268273

[20] Yung CF Kam KQ Nadua KD Chong CY Tan NWH Li J Novel coronavirus 2019 transmission risk in educational settings. Clin Infect Dis. 2021;72(6):1055-8. 10.1093/cid/ciaa794 32584975 PMC7337629

[21] Ismail SA, Saliba V, Bernal JL, Ramsay ME, Ladhani SN. SARS-CoV-2 infection and transmission in educational settings: cross-sectional analysis of clusters and outbreaks in England. London: Public Health England. 2020. Available from: https://assets.publishing.service.gov.uk/government/uploads/system/uploads/attachment_data/file/911267/School_Outbreaks_Analysis.pdf 10.1016/S1473-3099(20)30882-3 PMC7833602 33306981

[22] Link-Gelles R DellaGrotta AL Molina C Clyne A Campagna K Lanzieri TM Limited Secondary Transmission of SARS-CoV-2 in Child Care Programs - Rhode Island, June 1-July 31, 2020. MMWR Morb Mortal Wkly Rep. 2020;69(34):1170-2. 10.15585/mmwr.mm6934e2 32853185 PMC7451972

[23] Stein-Zamir C Abramson N Shoob H Libal E Bitan M Cardash T A large COVID-19 outbreak in a high school 10 days after schools’ reopening, Israel, May 2020. Euro Surveill. 2020;25(29):2001352. 10.2807/1560-7917.ES.2020.25.29.2001352 32720636 PMC7384285

[24] Torres JP Piñera C De La Maza V Lagomarcino AJ Simian D Torres B SARS-CoV-2 Antibody Prevalence in Blood in a Large School Community Subject to a COVID-19 Outbreak: A Cross-sectional Study. Clin Infect Dis. 2020;ciaa955. 10.1093/cid/ciaa955 32649743 PMC7454451

[25] Bernard Stoecklin S Rolland P Silue Y Mailles A Campese C Simondon A Investigation Team. First cases of coronavirus disease 2019 (COVID-19) in France: surveillance, investigations and control measures, January 2020. Euro Surveill. 2020;25(6):2000094. 10.2807/1560-7917.ES.2020.25.6.2000094 32070465 PMC7029452

[26] Fafi-Kremer S Bruel T Madec Y Grant R Tondeur L Grzelak L Serologic responses to SARS-CoV-2 infection among hospital staff with mild disease in eastern France. EBioMedicine. 2020;59:102915. 10.1016/j.ebiom.2020.102915 32747185 PMC7502660

[27] Grzelak L Temmam S Planchais C Demeret C Tondeur L Huon C A comparison of four serological assays for detecting anti-SARS-CoV-2 antibodies in human serum samples from different populations. Sci Transl Med. 2020;12(559):eabc3103. 10.1126/scitranslmed.abc3103 32817357 PMC7665313

[28] Sermet-Gaudelus I Temmam S Huon C Behillil S Gajdos V Bigot T Prior infection by seasonal coronaviruses, as assessed by serology, does not prevent SARS-CoV-2 infection and disease in children, France, April to June 2020. Euro Surveill. 2021;26(13):2001782. 10.2807/1560-7917.ES.2021.26.13.2001782 33797390 PMC8017906

[29] Wölfel R Corman VM Guggemos W Seilmaier M Zange S Müller MA Virological assessment of hospitalized patients with COVID-2019. Nature. 2020;581(7809):465-9. 10.1038/s41586-020-2196-x 32235945

[30] Santé publique France (SPF). COVID-19. Point épidémiologique - Situation au 5 mars 2020 - 15h. [COVID-19. Epidemiological update - Situation on March 5, 2020 - 3 p.m.]. Saint-Maurice: SPF; 2020. French. Available from: https://www.santepubliquefrance.fr/content/download/235532/2526871

[31] Menni C Valdes AM Freidin MB Sudre CH Nguyen LH Drew DA Real-time tracking of self-reported symptoms to predict potential COVID-19. Nat Med. 2020;26(7):1037-40. 10.1038/s41591-020-0916-2 32393804 PMC7751267

[32] Bénézit F Le Turnier P Declerck C Paillé C Revest M Dubée V RAN COVID Study Group. Utility of hyposmia and hypogeusia for the diagnosis of COVID-19. Lancet Infect Dis. 2020;20(9):1014-5. 10.1016/S1473-3099(20)30297-8 32304632 PMC7159866

[33] Buitrago-Garcia D Egli-Gany D Counotte MJ Hossmann S Imeri H Ipekci AM Occurrence and transmission potential of asymptomatic and presymptomatic SARS-CoV-2 infections: A living systematic review and meta-analysis. PLoS Med. 2020;17(9):e1003346. 10.1371/journal.pmed.1003346 32960881 PMC7508369

[34] Long QX Liu BZ Deng HJ Wu GC Deng K Chen YK Antibody responses to SARS-CoV-2 in patients with COVID-19. Nat Med. 2020;26(6):845-8. 10.1038/s41591-020-0897-1 32350462

[35] Sette A Crotty S. Pre-existing immunity to SARS-CoV-2: the knowns and unknowns. Nat Rev Immunol. 2020;20(8):457-8. 10.1038/s41577-020-0389-z 32636479 PMC7339790

[36] Anderson EM Goodwin EC Verma A Arevalo CP Bolton MJ Weirick ME UPenn COVID Processing Unit. Seasonal human coronavirus antibodies are boosted upon SARS-CoV-2 infection but not associated with protection. Cell. 2021;184(7):1858-1864.e10. 10.1016/j.cell.2021.02.010 33631096 PMC7871851

[37] Salje H Tran Kiem C Lefrancq N Courtejoie N Bosetti P Paireau J Estimating the burden of SARS-CoV-2 in France. Science. 2020;369(6500):208-11. 10.1126/science.abc3517 32404476 PMC7223792

