# Supplementary material for: SARS-CoV-2 infection in schools in a northern French city: a retrospective serological cohort study in an area of high transmission, France, January to April 2020
Source: Euro Surveill. 2021 Apr 15;26(15):2001695. doi: 10.2807/1560-7917.ES.2021.26.15.2001695 (PMC8167414; doi:10.2807/1560-7917.ES.2021.26.15.2001695)

Supplement S1:

SARS-CoV-2 infection in schools in a northern French city: a retrospective serological cohort study in an area of high transmission, France, January to April 2020

Arnaud Fontanet, Laura Tondeur, Rebecca Grant, Sarah Temmam, Yoann Madec, Thomas Bigot Ludivine Grzelak, Isabelle Cailleau, Camille Besombes, Marie-Noëlle Ungeheuer, Charlotte Renaudat, Blanca Liliana Perlaza, Laurence Arowas, Nathalie Jolly, Sandrine Fernandes Pellerin, Lucie Kuhmel, Isabelle Staropoli, Christèle Huon, Kuang-Yu Chen, Bernadette Crescenzo-Chaigne, Sandie Munier, Pierre Charneau, Caroline Demeret, Timothée Bruel, Marc Eloit, Olivier Schwartz, Bruno Hoen

Disclaimer

This supplementary material is hosted by *Eurosurveillance* as supporting information alongside the article 'SARS-CoV-2 infection in schools in a northern French city: a retrospective serological cohort study in an area of high transmission, France, January to April 2020,' on behalf of the authors, who remain responsible for the accuracy and appropriateness of the content. The same standards for ethics, copyright, attributions and permissions as for the article apply. Supplements are not edited by *Eurosurveillance* and the journal is not responsible for the maintenance of any links or email addresses provided therein.

Figure S1. Flowchart of enrolment of participants linked to the high school

Table S1. Characteristics of the 664 participants of the SARS-CoV-2 seroepidemiological investigation linked to a high school in a small city in northern France from 30 March to 4 April 2020

Table S2. Seropositivity according to symptoms and serological assay performed on serum samples from 664 participants linked to the high school

Figure S2. Flowchart of enrolment of participants linked to the primary schools

Table S3. Sociodemographic characteristics of the 1340 participants of the primary school investigation, in a city in northern France, 28-30 April 2020

Text: Further characteristics of infected symptomatic children in three separate schools

Figure S3: Presumable intrafamilial transmission based among family members linked to the primary schools based on date of symptom onset and symptom duration (shaded area) A. Family of a seropositive pupil who attends primary school C. B. Family of a seropositive pupil who attends primary school B.

Figure S1. Flowchart of enrolment of participants linked to the high school

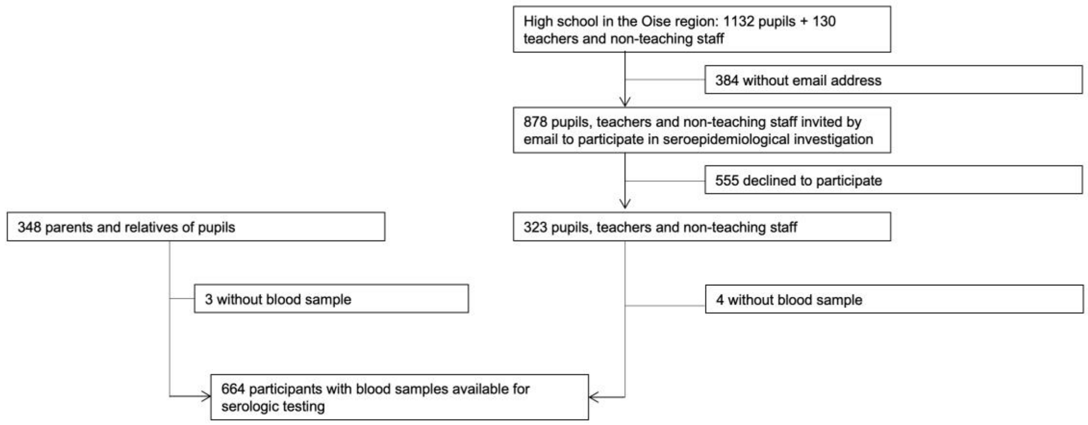

Table S1. Characteristics of the 664 participants of the SARS-CoV-2 seroepidemiological investigation linked to a high school in a small city in northern France from 30 March to 4 April 2020

	Teaching staff (n=53)	Non-teaching staff (n=27)	Pupils (n=239)	Parents of pupils (n=228)	Relatives of pupils (n=117)	Total (n=664)
Male gender	22 (41.5)	10 (37.0)	92 (38.5)	91 (39.9)	38 (32.5)	253 (38.1)
Age (years), Median (IQR)	43 (37-49)	51 (34-58)	16 (16-17)	47 (44-50)	25 (14-45)	38 (16-47)
Age groups						
≤14	0 (0)	0 (0)	3 (1.2)	0 (0)	34 (29.1)	37 (5.6)
15-17	0 (0)	0 (0)	204 (85.4)	0 (0)	2 (1.7)	206 (31.0)
18-44	30 (56.6)	11 (40.7)	32 (13.4)	58 (25.5)	46 (39.3)	177 (26.7)
45-64	23 (43.4)	16 (59.3)	0 (0)	168 (73.7)	34 (29.1)	241 (36.3)
≥65	0 (0)	0 (0)	0 (0)	1 (0.4)	1 (0.8)	2 (0.3)
Missing	0 (0)	0 (0)	0 (0)	1 (0.4)	0 (0)	1 (0.1)
Symptoms						
None	6 (11.3)	4 (14.8)	67 (28.0)	79 (34.7)	55 (47.0)	211 (31.8)
Minor only	8 (15.1)	6 (22.2)	44 (18.4)	50 (21.9)	23 (19.7)	131 (19.7)
Major	39 (73.6)	17 (63.0)	128 (53.6)	99 (43.4)	39 (33.3)	322 (48.5)

Detail of symptoms						
Fever	23 (43.4)	11 (40.7)	72 (30.1)	46 (20.2)	19 (16.2)	171 (25.7)
Cough	26 (49.1)	12 (44.4)	89 (37.2)	75 (32.9)	29 (24.8)	231 (34.7)
Dyspnea	13 (24.5)	2 (7.4)	27 (11.3)	37 (16.2)	11 (9.4)	90 (13.5)
Anosmia	17 (32.1)	7 (25.9)	19 (7.8)	11 (4.8)	4 (3.4)	58 (8.7)
Ageusia	14 (26.4)	6 (22.2)	20 (8.4)	11 (4.8)	7 (6.0)	58 (8.7)
Myalgia	24 (45.3)	14 (51.8)	50 (20.9)	54 (23.7)	14 (12.0)	156 (23.5)
Sore throat	16 (30.2)	9 (33.3)	75 (31.4)	56 (24.6)	20 (17.1)	176 (26.5)
Rhinitis	30 (56.6)	15 (55.6)	103 (43.1)	62 (27.2)	31 (26.5)	241 (36.3)
Diarrhea	11 (20.7)	7 (25.9)	36 (15.1)	25 (11.0)	13 (11.1)	92 (13.9)
Headache	28 (52.8)	11 (40.7)	75 (31.4)	58 (25.4)	31 (26.5)	203 (30.6)
Asthenia	27 (50.9)	10 (37.0)	60 (25.1)	63 (27.6)	29 (24.8)	189 (28.5)
Other	17 (32.1)	4 (14.8)	35 (14.6)	28 (12.3)	5 (4.3)	89 (13.4)

Table S2. Seropositivity according to symptoms and serological assay performed on serum samples from 664 participants linked to the high school

	Total n=664 (%)	No symptoms n=211 (%)	Minor only n=131 (%)	Major n=322 (%)
S-Flow	164 (24.7)	24 (11.4)	20 (15.3)	120 (37.3)
ELISA	96 (14.5)	12 (5.7)	10 (7.6)	74 (23.0)
LIPS	132 (19.9)*	17 (8.1)*	15 (11.4)	100 (31.1)
Any positive	167 (25.1)	24 (11.4)	21 (16.0)	122 (37.9)
Combination of assays				
None positive	497 (74.8)	187 (88.6)	110 (84.0)	200 (62.1)
1 positive	31 (4.7)	7 (3.3)	6 (4.6)	18 (5.6)
2 positives	47 (7.1)	5 (2.4)	6 (4.6)	36 (11.2)
3 positives	89 (13.4)	12 (5.7)	9 (6.9)	68 (21.1)

*1 sample with no result

Figure S2. Flowchart of enrolment of participants linked to the primary schools

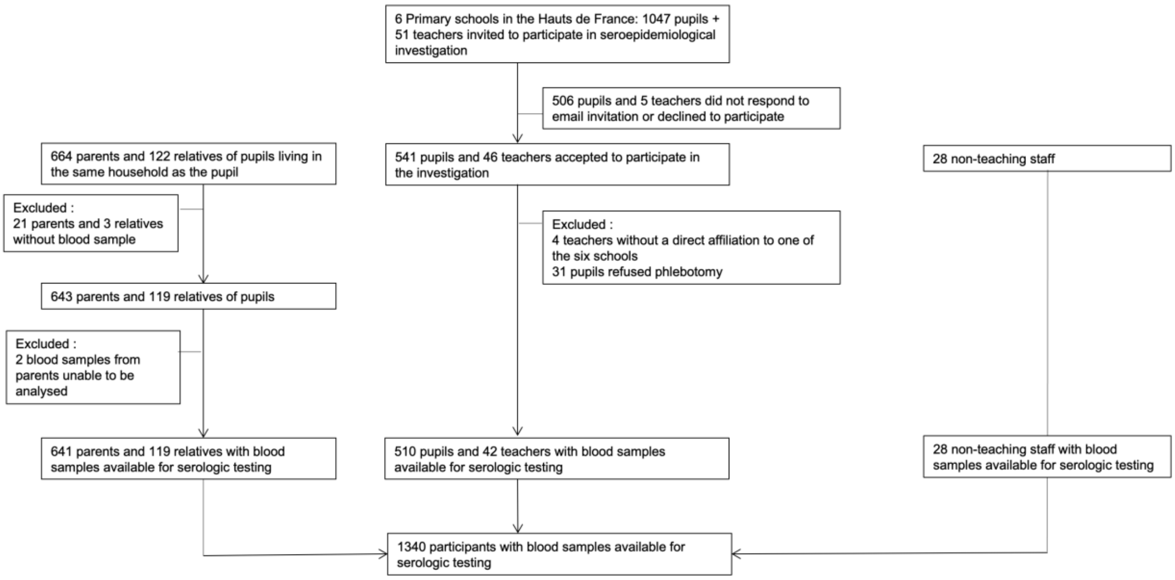

Table S3. Sociodemographic characteristics of the 1340 participants of the primary school investigation, in a city in northern France, 28-30 April 2020

	Teaching staff n=42 (%)	Non-teaching staff n=28 (%)	Pupils n=510 (%)	Parents n=641 (%)	Relatives n=119 (%)	Total n=1340 (%)
Sex						
Male	4 (9.5)	3 (10.7)	259 (50.8)	252 (39.3)	53 (44.5)	571 (42.6)
Age (in years)						
≤ 7	0 (0)	0 (0)	152 (29.8)	0 (0)	9 (7.6)	161 (12.0)
8-9	0 (0)	0 (0)	203 (39.8)	0 (0)	2 (1.7)	205 (15.3)
10-11	0 (0)	0 (0)	155 (30.4)	0 (0)	18 (15.1)	173 (12.9)
12-17	0 (0)	0 (0)	0 (0)	0 (0)	78 (65.6)	78 (5.8)
18-44	19 (45.2)	12 (42.9)	0 (0)	501 (78.2)	10 (8.4)	542 (40.5)
45-64	23 (54.8)	16 (57.1)	0 (0)	138 (21.5)	2 (1.7)	179 (13.4)
≥ 65	0 (0)	0 (0)	0 (0)	2 (0.3)	0 (0)	2 (0.1)
School						
A	5 (11.9)	10 (35.7)	61 (12.0)	67 (10.4)	20 (16.8)	163 (12.2)
B	5 (11.9)	7 (25.0)	90 (17.6)	108 (16.8)	18 (15.1)	228 (17.0)
C	6 (14.3)	4 (14.3)	68 (13.3)	78 (12.2)	15 (12.6)	171 (12.8)
D	8 (19.0)	4 (14.3)	87 (17.1)	116 (18.1)	24 (20.2)	239 (17.8)
E	11 (26.2)	0 (0.0)	117 (22.9)	151 (23.6)	25 (21.0)	304 (22.7)
F	7 (16.7)	3 (10.7)	87 (17.1)	121 (18.9)	17 (14.3)	235 (17.5)

Text: Further characteristics of infected symptomatic children in three separate schools

In the first instance (Figure S3A), a boy aged 10 complained of acute gastroenteritis on 17 January, with spontaneous resolution over the weekend and he returned to school C on 20 January despite some tiredness. On 3 February, he reported fever (38°C), cough, nausea and vomiting, and fatigue. He attended school the entire week, but did not go to his judo classes. He continued to feel tired and breathless when playing with friends during recess. His 16-year-old brother reported a sore throat between 20-23 January. He returned to high school on 28 January, but missed class on 29 and 30 January, and reported fever, nausea, vomiting and diarrhea on 3 February. He did not attend classes on 5-6 February, and returned to school on 7 February until the school holidays (14 February). His serology result was positive. His 11-year-old sister who attends middle school reported nausea, vomiting, and fatigue between 22 January and 3 February. Her serology result, done in a local laboratory, was negative. On 14 February, his mother reported fever, cough, and extreme fatigue, for four days. She continued to complain of breathing difficulties. Her serology result was positive. The father never reported any illness and was not tested for SARS-CoV-2 antibodies.

In the second instance (Figure S3B), an 8-year-old girl started complaining of extreme fatigue at school B on 11 February. She developed fever the following day (38°C), a runny nose, and spent the day with a caregiver. She returned to school on 13 and 14 February, and was diagnosed with rhinopharyngitis by her general practioner on 14 February. She developed a cough on 14 February and diarrhea on 18 February. Her 16-year-old brother, who attends the high school where the epidemic was very active during the first two weeks of February, complained of headache, fatigue, and stuffy nose from 7 February. He developed anosmia and ageusia the following week, and had a positive serology result for SARS-CoV-2. Her 15-year-old brother reported fatigue and ageusia, on 15 February and also had a positive serology result for SARS-CoV-2. On 21 February, her mother reported fever, nausea, and extreme fatigue, followed by diarrhea on 23 February, and had a positive serology result for SARS-CoV-2. On 24 February, the father reported fatigue and myalgia, followed by anosmia and ageusia on 27 February and had a positive serology result for SARS-CoV-2.

In the third instance, a girl, aged 10, complained of fever (39°C), sore throat, headache, nausea and abdominal pain on 9 February. Fever lasted three days, and was followed by rhinorrhea and diarrhea for

two days. She stayed at home and returned to school E on 14 February, although she continued to complain of fatigue for several weeks. Her 22-year-old sister aged and her mother reported feeling unwell during the same time period, but they both had a negative serology result for SARS-CoV-2.

Figure S3: Symptoms history among family members linked to the primary schools cases based on date of symptom onset. Shaded areas indicate days with symptoms, and dashed shaded areas days where the child was symptomatic and at school.

A. Family of a seropositive pupil who attends primary school C

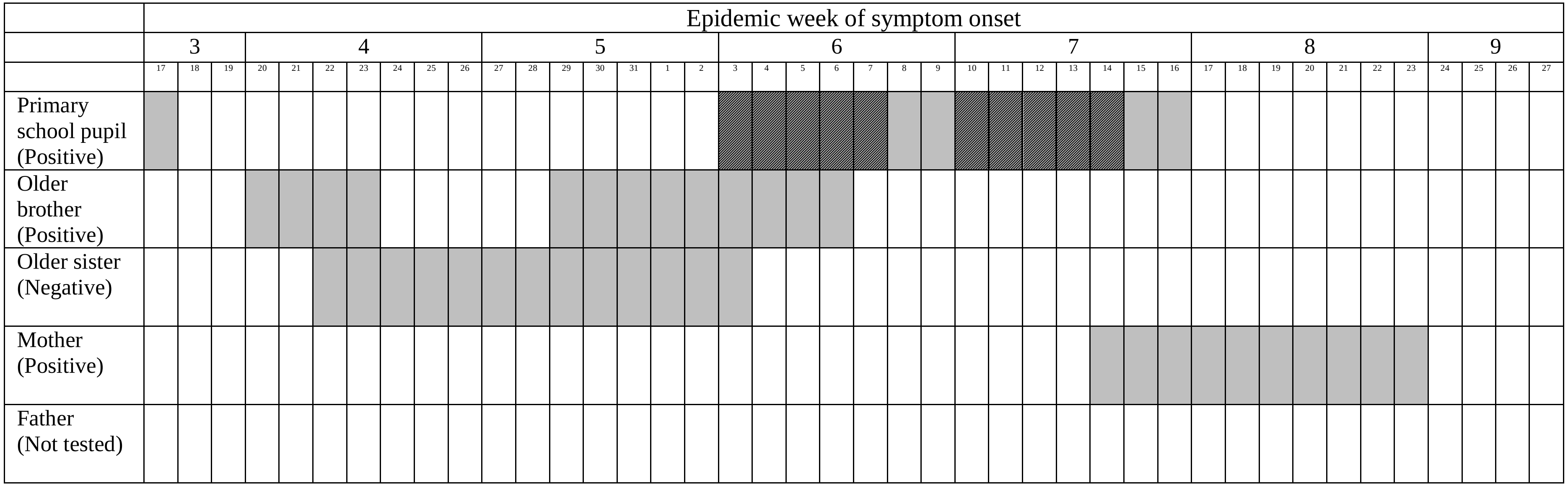

B. Family of a seropositive pupil who attends primary school B

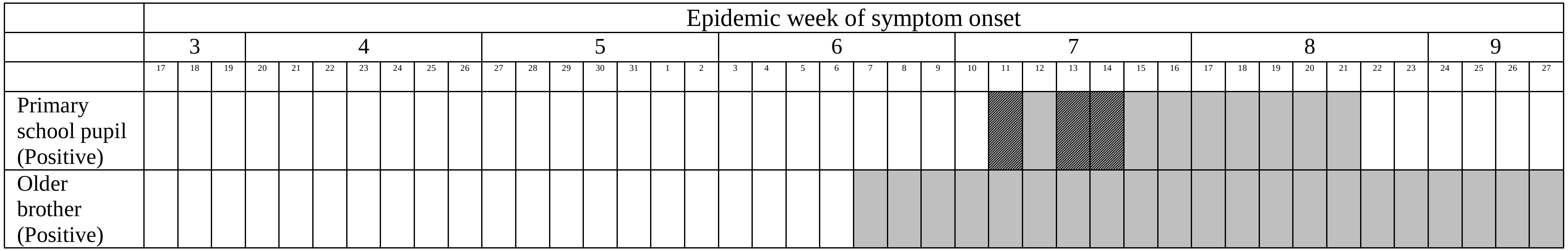

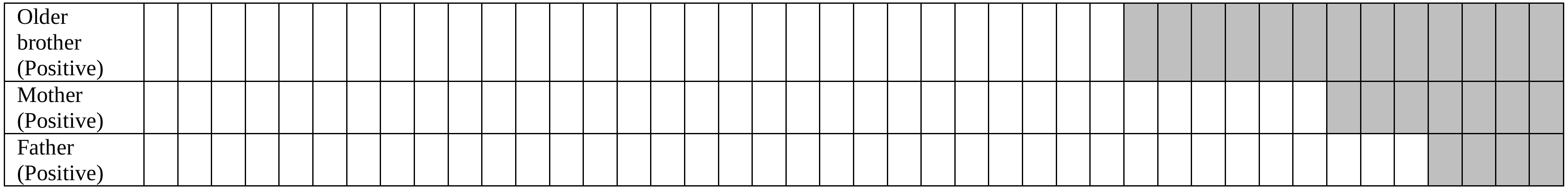

C. Family of a seropositive pupil who attends primary school E

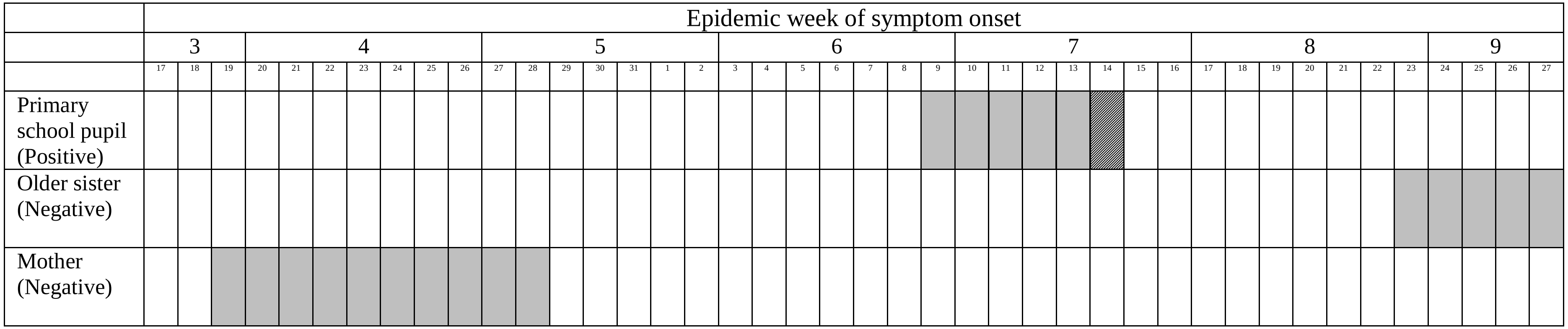

Supplement: Supplementary Material [file 2001695_SupplementaryMaterial.pdf]
